# Temporal trends in semen parameters among men attending a fertility center in the UAE (2012 – 2022): a retrospective study

**DOI:** 10.3389/fphys.2026.1801736

**Published:** 2026-06-10

**Authors:** Temidayo S. Omolaoye, Jeyaseelan Lakshmanan, Amin Abu Hijleh, Irfan Aslam, Stefan S. Du Plessis

**Affiliations:** 1 College of Medicine, Mohammed Bin Rashid University of Medicine and Health Sciences, Dubai Health, Dubai, United Arab Emirates; 2 Research and Graduate Studies, Mohammed Bin Rashid University of Medicine and Health Sciences, Dubai Health, Dubai, United Arab Emirates; 3 IVF Lab, HealthPlus Fertility Center, Abu Dhabi, United Arab Emirates

**Keywords:** age, body mass index, Emirati men, environmental stress, male fertility, Middle East, semen parameters, sperm motility

## Abstract

**Background:**

Decline in semen quality have been widely debated for decades, however, most global assessments lack data from the Middle East. To address this gap, this study characterized eleven-year temporal trends in semen parameters among Emirati men, accounting for the effects of age and body mass index (BMI).

**Methods:**

A retrospective longitudinal analysis was conducted using 13,276 semen samples collected from 2,730 Emirati men attending a fertility center in the United Arab Emirates between 2012 and 2022. Semen parameters, including semen volume, sperm concentration, total sperm count (TSC), progressive motility, and total motility, were analyzed for temporal trends and stratified by age and BMI using generalized estimating equation (GEE) modelling. Yearly differences were assessed using ANOVA with Bonferroni or Tukey’s *post-hoc* tests.

**Results:**

All measured semen parameters remained above the World Health Organization lower reference limits, but significant year-to-year fluctuations in key measures were observed. A pronounced dip in semen volume, sperm concentration, and TSC occurred in 2016, with partial recovery in subsequent years. In contrast, both progressive and total sperm motility declined steadily throughout the decade, with yearly decreases of 6% to 17%. Age above 35 years was consistently associated with declines in volume and motility, and higher BMI was linked to reduced semen quality, though the rate of decline over time did not differ significantly by BMI.

**Conclusions:**

Temporal changes in semen parameters among Emirati men visiting a fertility clinic echoes trends observed globally but is shaped by unique demographic and environmental factors of the region. These findings underscore the complexity of male fertility trends, and it is recommended that studies integrating environmental, lifestyle, and demographic data to understand reproductive status in the Middle East be conducted.

## 1 Introduction

The discussion about the temporal decline in semen quality started more than nine decades ago (Hotchkiss et al., 1938), but became reiterative with the study of Carlsen et al (Carlsen et al., 1992), who reported a decrease in the average sperm count from 113×10^6^/ml in 1940 to 66×10^6^ in 1990. However, in 1995, Olsen et al. re-evaluated the 61 studies examined by Carlsen et al. using a stairstep model rather than linear regression and reported a decline in sperm count during the 1960s, after which the mean sperm count remained constant (Olsen et al., 1995). They further argued that, among the four models assessed, quadratic, spline fit, stairstep, and linear, the stairstep model provided the most accurate and biologically plausible representation of the data at both ends of the study period.

Based on the partial contradiction in findings, Olsen et al. raised the following questions: has there been a real decline in mean sperm count? If yes, why? One of the main limitations identified by Olsen et al. was that 48 of the 61 studies (78.7%) included in Carlsen et al.’s analysis were published after 1970, indicating that the findings primarily reflect the period between 1970 and 1991 rather than earlier decades. Furthermore, of the 14,947 men constituted in the 61 studies, only 596 men were before 1950, and 13,167 men since 1970, further justifying the skewness in data.

To shed light on this inconsistency, Brake and Krase re-evaluated the 48 studies published between 1970 and 1991 (Brake and Krause, 1992). They reported small but statistically significant increase in mean sperm concentration and recommended that their findings should not be overvalued and interpreted with caution. However, they mentioned that historical values of mean sperm concentration between 1938 and 1969 were significantly higher than those observed between 1970 and 1990.

With the ongoing debate as to whether there is indeed a decline in sperm quality and quantity comes the study of Levine et al. first published in 2017, and an update published in 2023 (Levine et al., 2017, 2023). The authors reported a significant reduction in sperm count between 1973 and 2011, averaging 0.70 million/ml per year. They found an annual decrease of 1.4% in sperm concentration and 1.6% in total sperm count across North America, Europe and Australasia, amounting to overall declines of 52.4% and 59.3%, respectively. Although, no significant reductions were observed in studies from South America, Asia, and Africa, partly due to insufficient data. However, when the analysis accounted for geographic differences, a significant downward trend emerged in these regions as well, although the decline was less pronounced than in North America, Europe and Australasia.

In all the data presented, the trend in semen parameters among men of Middle Eastern heritage is not accounted for. To have cumulative evidence of what is going on globally, it is pertinent to observe data coming from different countries over a certain period. That is, looking at the data country-wise before moving to region and subsequently, world-wide.

A study reported decline in semen parameters, specifically total motility, and increased frequency of azoospermia among men who visited a fertility clinic in Dhaka Bangladesh between 2000 and 2016 (Mahmud et al., 2018). Another study reported that semen parameters of Indian men have declined over time, and the deterioration is quantitatively higher in the infertile group (Mishra et al., 2018). In the same vein, a study from China showed that semen parameters, including motility and sperm count declined in men visiting a fertility centre over a seven-year period (Li et al., 2023). Several other studies from different countries including Nigeria and South Africa (Akang et al., 2023), United States (Chen et al., 2003; Miller et al., 2024), have reported decline in semen quality over the years. Although the studies cited above had different study population characteristics, what seem to cross through, is a decline in sperm motility. If this is because of increasing age in these populations, remains to be elucidated.

Recognizing that existing global assessments of semen quality are not fully representative of all regions of the world, and that Middle Eastern data remain notably underrepresented, this study examined ten-year trends in semen parameters among Emirati men attending a fertility center in the United Arab Emirates.

## 2 Methods

### 2.1 Data acquisition

Ethical approvals were obtained from the Mohammed Bin Rashid University of Medicine and Health Sciences (MBRU) Ethics Committee (MBRU-IRB-2021-12) and HealthPlus Research and Ethics Committee (REC/2022/P27). Data were extracted from the fertility center’s database and stored on password protected secured server. Data collection, filtering and vetting, and unadjusted data analysis were performed as described in our previous study (Omolaoye et al., 2024).

### 2.2 Participants

The anthropometric information including age, weight, height, body mass index (BMI), and nationality, along with semen parameters (semen volume, sperm concentration, TSC, total motility, progressive motility) of Emirati men who attended a Fertility Center in Abu Dhabi, between 2012 and July 2022 were assessed. Semen parameters were analysed based on the 5^th^ edition of the World Health Organization (WHO) laboratory manual for the examination and processioning of human semen (World Health Organization, 2010). The study adheres to standard laboratory procedures and quality control measures.

Since the current study focuses exclusively on Emirati men, only samples collected from this population were included in the analysis.

This study forms part of a larger ongoing project from which our previous study (Omolaoye et al., 2024) also originated, involving 32,664 samples collected from patients across 113 different countries. From this cohort, the 21,651 samples obtained from UAE nationals formed the starting samples for the current study.

**Figure 1:**
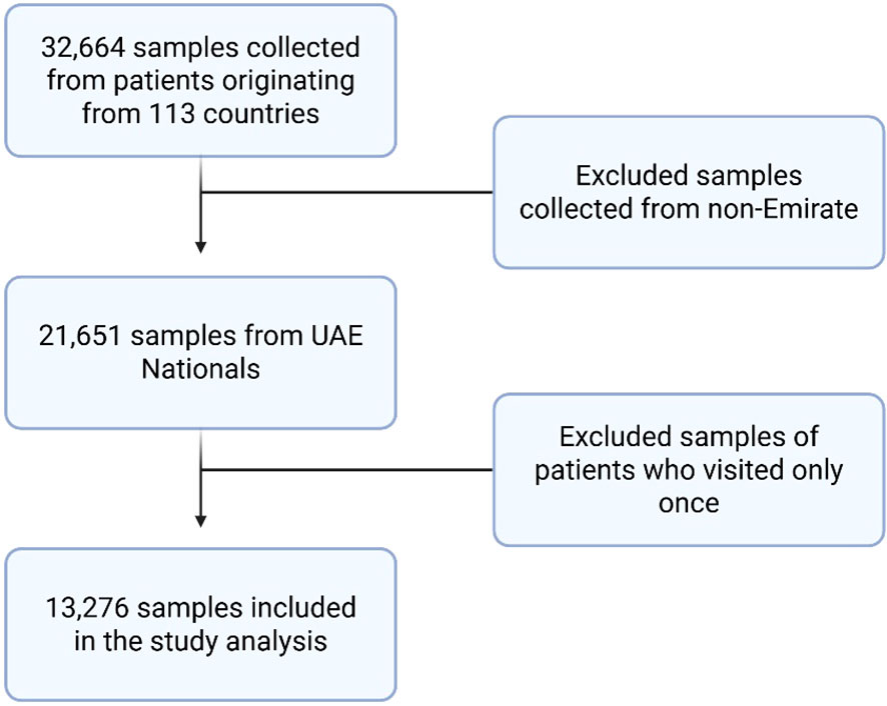
Flow chart representation of data included in the study.

To evaluate the repetitive measure of semen parameters for the same patient, thus allowing for trend evaluation, samples of patients who visited only once were excluded from the analysis. After exclusion, 13,276 samples remained, representing 2,730 patients of Emirate origin (**Figure 1**).

### 2.3 Statistical analysis

Data were analyzed using SPSS (IBM SPSS, v29.0, USA) and GraphPad Prism™ (GraphPad™ Software, Version 10.2.3, San Diego, CA, USA). The regression lines with 95% CI band over time by covariates for various semen parameters were performed using R codes (version 4, The R Foundation for Statistical Computing Platform). As the observations were repeated over time, the regression models for longitudinal data analyses (Generalized Estimating Equation (GEE)) were performed. The GEE method was used because it is appropriate for longitudinal data analyses, which has one hierarchical (higher) level, that is, study subjects. When the correlation structure in the GEE model is Exchangeable, then both hierarchical and GEE models will provide the same regression coefficients. The covariates used were age and BMI. However, the significance of a covariate was assessed by the interaction term between time and the covariate. The study outcomes include semen volume, sperm concentration, TSC, total motility and progressive motility.

The study also employed both unadjusted and adjusted models to examine associations between semen parameters and relevant variables. The adjusted analysis specifically investigated how semen parameters (sperm volume, concentration, total sperm count, and progressive and total motility) changed over 11-year period in an Emirati cohort, and how these trends were associated with age and BMI.

For the temporal trends descriptive and comparison analysis, a one-way Anova and Bonferroni and Tukey’s comparison tests were used. A p< 0.05 was considered statistically significant.

## 3 Results

### 3.1 Description of study population

To evaluate the temporal trends in semen parameters of UAE nationals over a decade, semen samples from UAE nationals were identified and stratified from a larger dataset of 32,664 samples, which had been collected over an 11-year period from men representing 113 different countries. The demographic characteristics of the entire study cohort have been described in our previous study (Omolaoye et al., 2024).

From the initial pool of 32,664 semen samples, all non-Emirati samples were excluded, resulting in 21,651 samples from UAE nationals. To enable the analysis of longitudinal trends, we further excluded samples from patients who had only a single clinic visit. The final analytical cohort consisted of 13,276 samples collected from 2730 patients with repeated measures. The average number of visits per patient was 5. The highest recorded number of visits for a single patient was 32. The age distribution at test is shown in **Table 1**.

**Table 1:** Age distribution of the subjects at test.

Age range	Frequency	Percent	Valid percent	Cumulative percent
<20	5	0.2	0.2	0.2
20-29.9	730	26.7	26.7	26.9
30-39.9	1205	44.1	44.2	71.1
40-49.9	597	21.9	21.9	93.0
50-59.9	142	5.2	5.2	98.2
60-69.9	50	1.8	1.8	100.0
Total	2729	100.0	100.0	
Missing System	1	.0		
Total	2730	100.0		

### 3.2 Description of semen parameters

A descriptive overview of the semen characteristics of Emirati men for the entire study period is presented in **Table 2**. The mean semen volume for the cohort was 2.51 ± 1.48 mL. The mean sperm concentration was 63.86 ± 56.26 × 10^6^/mL, with the 25th and 75th percentiles at 20 × 10^6^/mL and 90 × 10^6^/mL, respectively. The resulting mean TSC was 159.38 ± 175.25 ×10^6^.The mean progressive motility was 39.12 ± 19.66%, and the average total motility was 47.05 ± 20.02%.

**Table 2:** Description of semen parameters.

Parameters	N	Mean	Median	Std. deviation	Percentile	
					25	75
Volume (mL)	13276	2.51	2.00	1.48	1.50	3.00
Sperm Concentration (M/mL)	12212	63.86	52.00	56.26	20.00	92.00
Total Sperm Count (10^6^)	12195	159.38	108.00	175.25	36.00	222.50
Progressive Motility (%)	12046	39.12	39.00	19.66	25.00	53.50
Total Motility (%)	12023	47.05	47.50	20.02	33.00	61.50

Following the descriptive overview, individual semen parameters were analyzed for trends across the 11-year study period.

#### 3.2.1 Semen volume: temporal trends

Through the 11 years period, the number of samples evaluated per year ranged between 50 and 1996. Analysis revealed significant year-to-year variations in semen volume (p<0.01).

**Figure 2:**
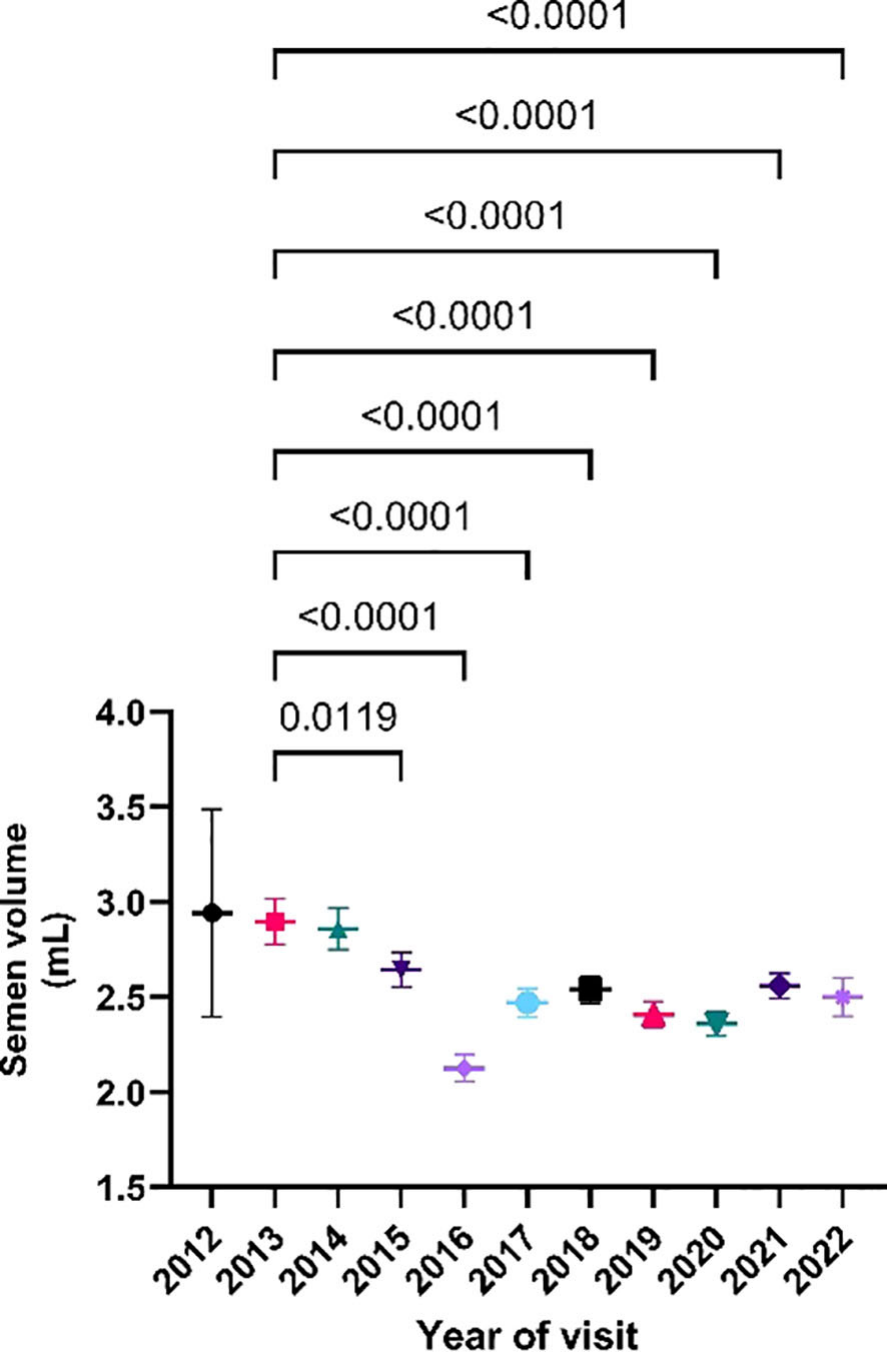
Temporal trend for semen volume by year of visit in Emirati cohort. A steep decline occurred in 2016, where volume reduced by approximately 0.4 mL between 2016 and 2022 (p<0.05). Although values stabilized post-2016, they remained below pre-decline levels throughout the remaining study period.

For instance, in 2013, semen volume was significantly higher compared to 2015 (mean difference: 0.252 mL; p<0.015); 2016 (mean difference: 0.771 mL; p<0.001); 2017 (mean difference: 0.426 mL; p<0.001), and through to 2022 (p<0.001) (**Figure 2**; **Supplementary Table 1**). Notably, a significant decrease in mean volume was observed in 2016 compared to preceding years.

#### 3.2.2 Sperm concentration and total sperm count: temporal trends

**Figure 3:**
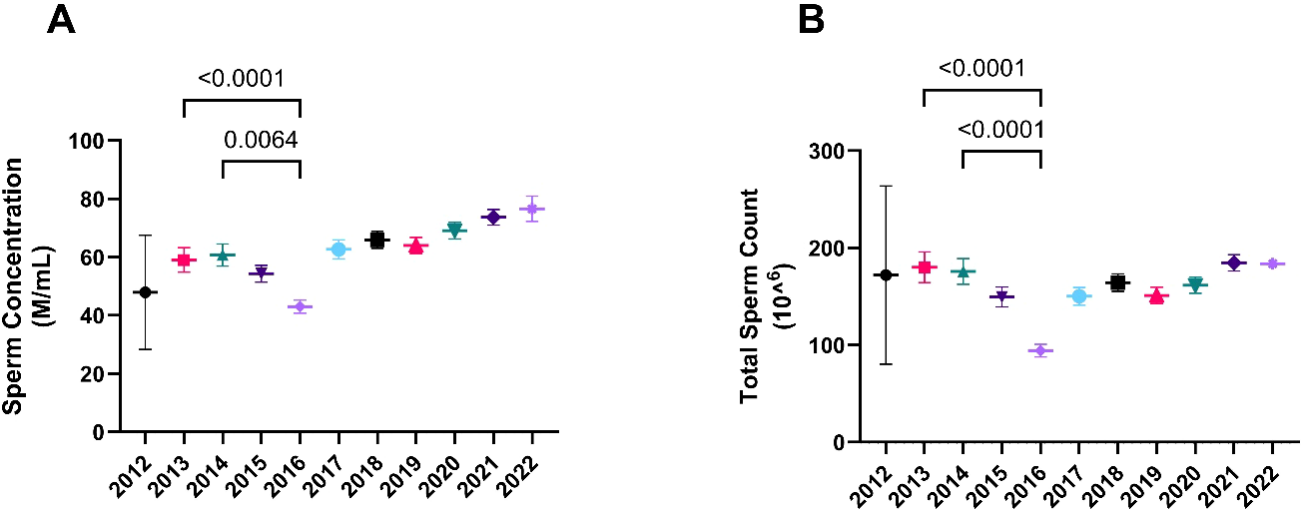
Temporal trend for sperm concentration and total sperm count by year of visit in Emirati cohort. **(A)** Sperm concentration by year; **(B)** Total sperm count by year. Both parameters significantly reduced in 2016 but improved in subsequent years, stabilizing at higher levels by 2022.

Sperm concentration significantly reduced in 2016 compared to the previous years (p<0.05), and thereafter consistently increased per year (**Figure 3A**; **Supplementary Table 2**). The observed average increase per year was 13.9 ×10^6^/mL between 2017 and 2022.

Similarly, there was a significant decrease in TSC in 2016 compared to the previous years, followed by a steady recovery in subsequent years (**Figure 3B**), thus showing the U-shaped” trajectory as seen in sperm concentration. These findings suggest temporal fluctuations in semen quantity with a notable dip in 2016, followed by recovery.

#### 3.2.3 Progressive motility and total motility: temporal trends

**Figure 4:**
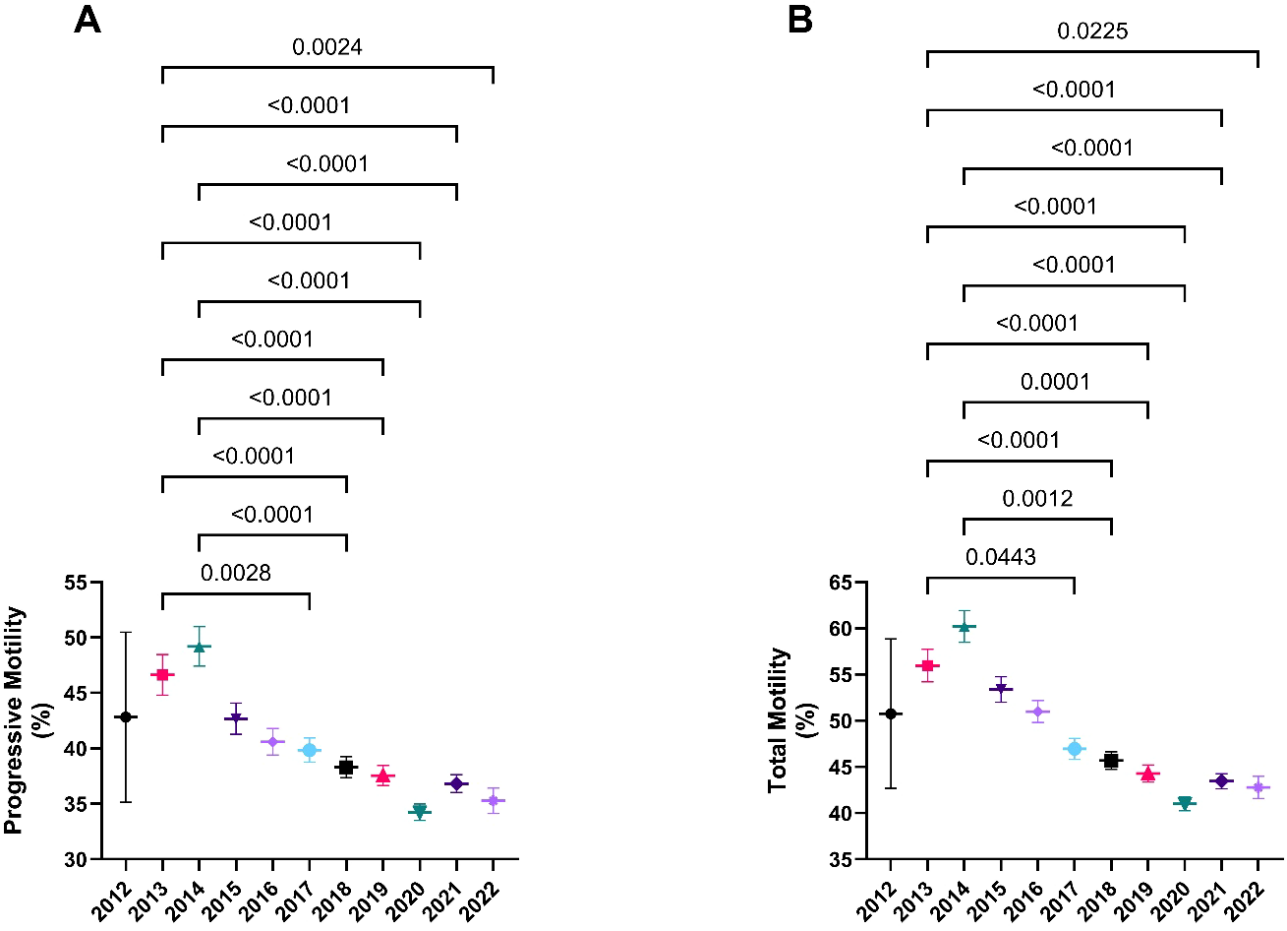
Temporal trend for sperm motility by year of visit in Emirati cohort. **(A)** Progressive motility by year; **(B)** Total motility by year. Significant year-to-year differences were observed, particularly after 2014, indicating a downward trend in sperm motility over time. This suggests a progressive reduction in semen quality despite improvements in quantity.

In contrast to the trends observed for sperm concentration and TSC, both progressive and total motility exhibited a consistent and significant decline from 2014 to 2020 (**Figures 4A, B**; **Supplementary Table 3**). The yearly decrease in motility ranged from 6% to 17%.

Despite the persistent decline observed in sperm motility and the fluctuations seen in sperm concentration and TSC, the mean values for all parameters remained above the WHO lower reference limits throughout the study period.

### 3.3 Effect of age and BMI on semen parameters

To further elucidate the semen characteristics of our Emirati cohort, we evaluated the impact of two key variables over 100 months: age and body mass index (BMI). Samples were stratified into two age groups [≤ 35 years; >35 years] and three BMI categories [Normal (18.5–24.9 kg/m²); Overweight (25.0–29.9 kg/m²); Obese (≥30 kg/m²)].

#### 3.3.1 Semen volume

**Figure 5:**
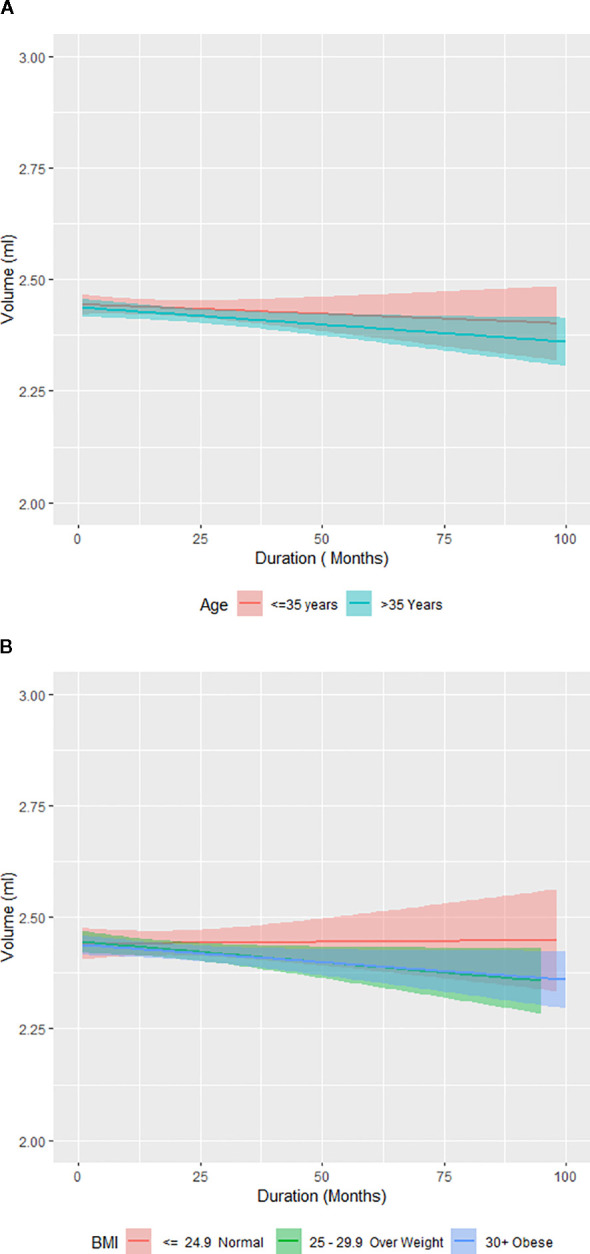
Effect of age and BMI on semen volume over 100 months. **(A)** Age and semen volume; **(B)** BMI and semen volume. The plot shows a gradual decline in semen volume over time in both age groups, with men >35 years exhibiting a slightly steeper reduction compared to younger men. Although the overall decrease is modest, the trend suggests age-related differences in semen volume decline across the follow-up period. Men with a normal BMI maintained the highest semen volume throughout the study, while obese and overweight participants exhibited the lowest values, suggesting an inverse relationship between BMI and semen volume across the 100-month observation period.

A consistent difference in semen volume was observed between age groups. The mean volume was significantly lower in the older age group (>35 years) compared to the younger group (≤35 years) (2.6 ± 1.5 mL vs. 2.4 ± 1.5 mL; p<0.01). However, as shown in **Figure 5A**, the longitudinal trend of semen volume over 100 months of follow up did not differ significantly between the two groups (**Table 3**; p = 0.610). This indicates that while older men presented with lower volumes overall, the rate of change over time was similar in both groups.

**Table 3:** Regression analyses for the effect of age and BMI on semen volume.

Parameters	Regression coefficient	95% confidence interval		P value
		Lower	Upper	
Intercept	2.984	2.816	3.152	<.001
Age	-.197	-.263	-.131	<.001
BMI	-.048	-.091	-.005	.030
Age * Duration (months)	-.001	-.003	.002	.610
BMI * Duration (months)	.000	-.002	.001	.876
Duration (months)	.001	-.006	.007	.814

A similar pattern was observed across BMI categories. Mean semen volume was lower in the overweight and obese groups compared to the normal BMI group (2.6 ± 1.5 mL vs. 2.5 ± 1.5 mL vs. 2.5 ± 1.5 mL; p=0.03). However, as shown in **Figure 5B**, longitudinal trends over the decade did not differ significantly among the three groups (**Table 3**; p=0.876). This indicates that although overweight and obese men had lower semen volumes overall, the rate of decline over time was comparable across BMI groups.

#### 3.3.2 Sperm concentration

**Figure 6:**
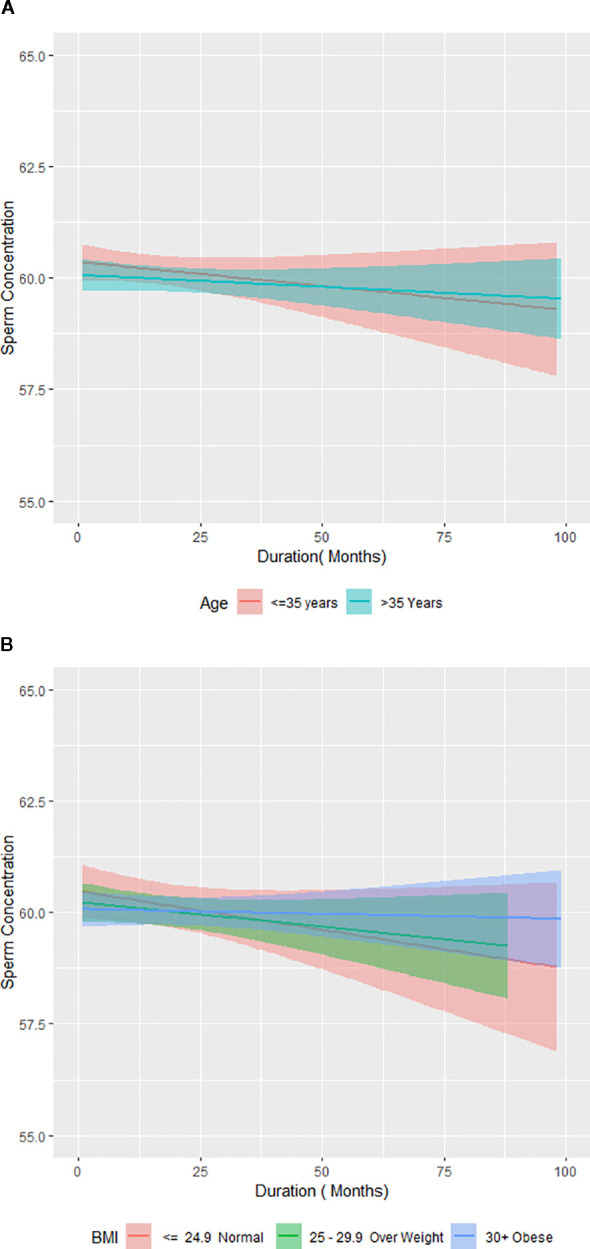
Effect of age and BMI on sperm concentration over 100 months. **(A)** Age and sperm concentration; **(B)** BMI and sperm concentration.

The mean sperm concentration was significantly higher in the >35 years group compared to the ≤35 years group (68.2 ± 58.3 vs. 58.1 ± 52.9 ×10^6^/mL; p<0.01). However, when analyzed longitudinally, no significant trend was observed within either age group (**Figure 6A**, **Table 4**).

**Table 4:** Regression analyses for the effect of age and BMI on sperm concentration.

Parameter	Regression coefficient	95% confidence interval		P value
		Lower	Upper	
Intercept	62.455	55.321	69.588	<.001
Age	13.324	10.526	16.122	<.001
BMI	-5.595	-7.505	-3.684	<.001
Duration (months)	.026	-.260	.312	.860
Age * Duration (months)	-.086	-.194	.022	.117
BMI * Duration (months)	.018	-.051	.087	.612

For BMI, sperm concentration was highest in men with a normal BMI, followed by overweight and obese men (67.9 ± 59.4 vs 66.4 ± 57.0 vs 60.1 ± 54.1 x 10^6^/mL; p<0.001). This pattern remained consistent across the study period, with no significant interaction between BMI categories and time (p = 0.612) (**Figure 6B**, **Table 4**).

#### 3.3.3 Total sperm count

**Figure 7:**
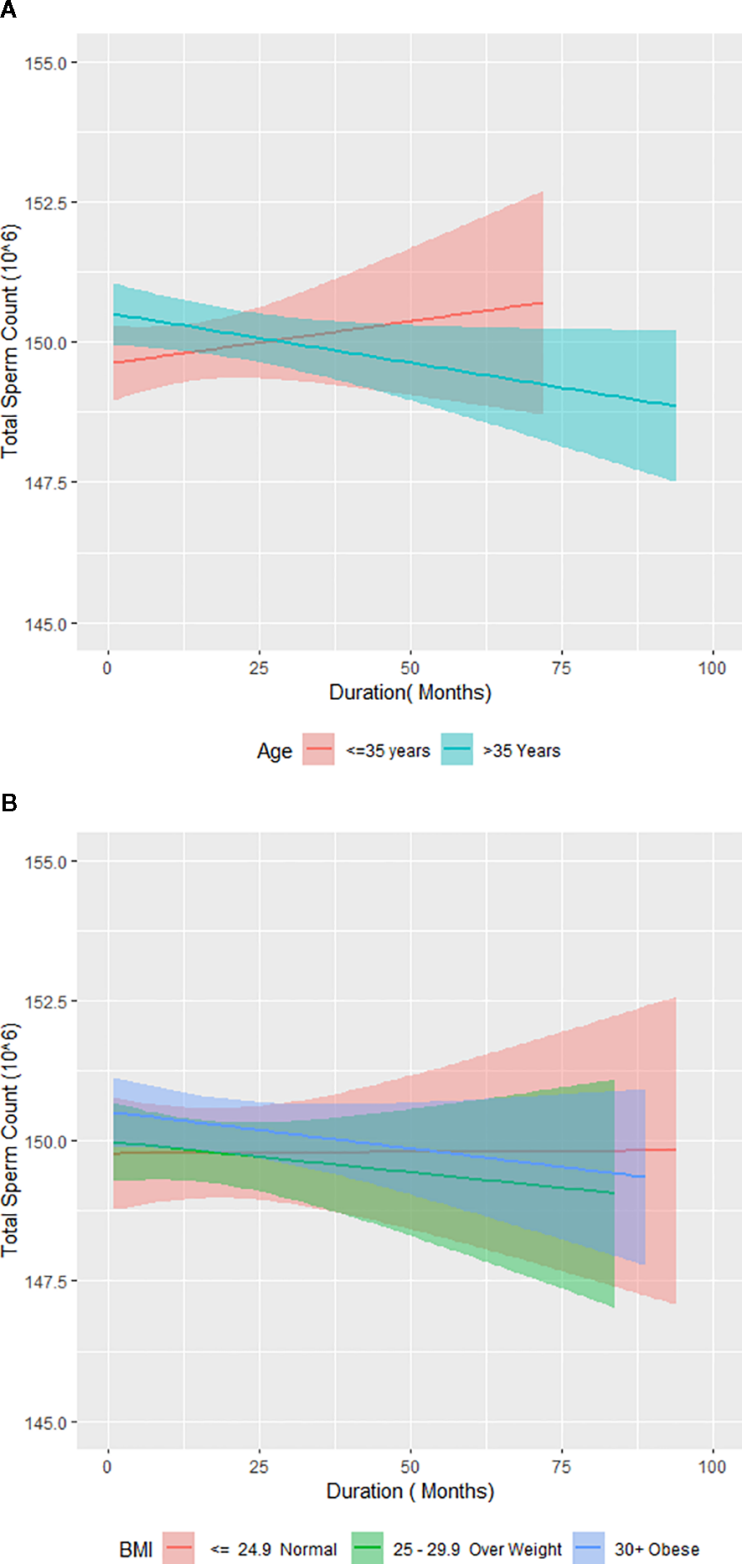
Effect of age and BMI on total sperm count over 100 months. **(A)** Age and TSC; **(B)** BMI and TSC.

As seen for sperm concentration, similar pattern was observed for TSC. The mean TSC was higher in the older age group compared to the younger group. While a non-significant increasing trend was observed in the ≤35 years group and a non-significant decreasing trend in the >35 years group over time (**Figure 7A**, **Table 5**).

**Table 5:** Regression analyses for the effect of age and BMI on total sperm count.

Parameter	Regression coefficient	95% confidence interval		P value
		Lower	Upper	
Intercept	172.775	149.630	195.920	<.001
Age	22.128	13.279	30.978	<.001
BMI	-13.154	-19.171	-7.137	<.001
Duration (months)	.296	-.612	1.205	.523
Age * Duration (months)	-.288	-.631	.054	.099
BMI * Duration (months)	-.017	-.236	.201	.876

Additionally, TSC was significantly influenced by BMI, with the highest values in the normal weight group and the lowest in the obese group (173.1 ± 190.3 vs. 165.9 ± 175.0 vs. 148.4 ± 168.3 ×10^6^; p<0.001) (**Figure 7B**, **Table 5**). No significant temporal trend was observed across BMI categories (p = 0.876).

#### 3.3.4 Progressive and total motility

**Figure 8:**
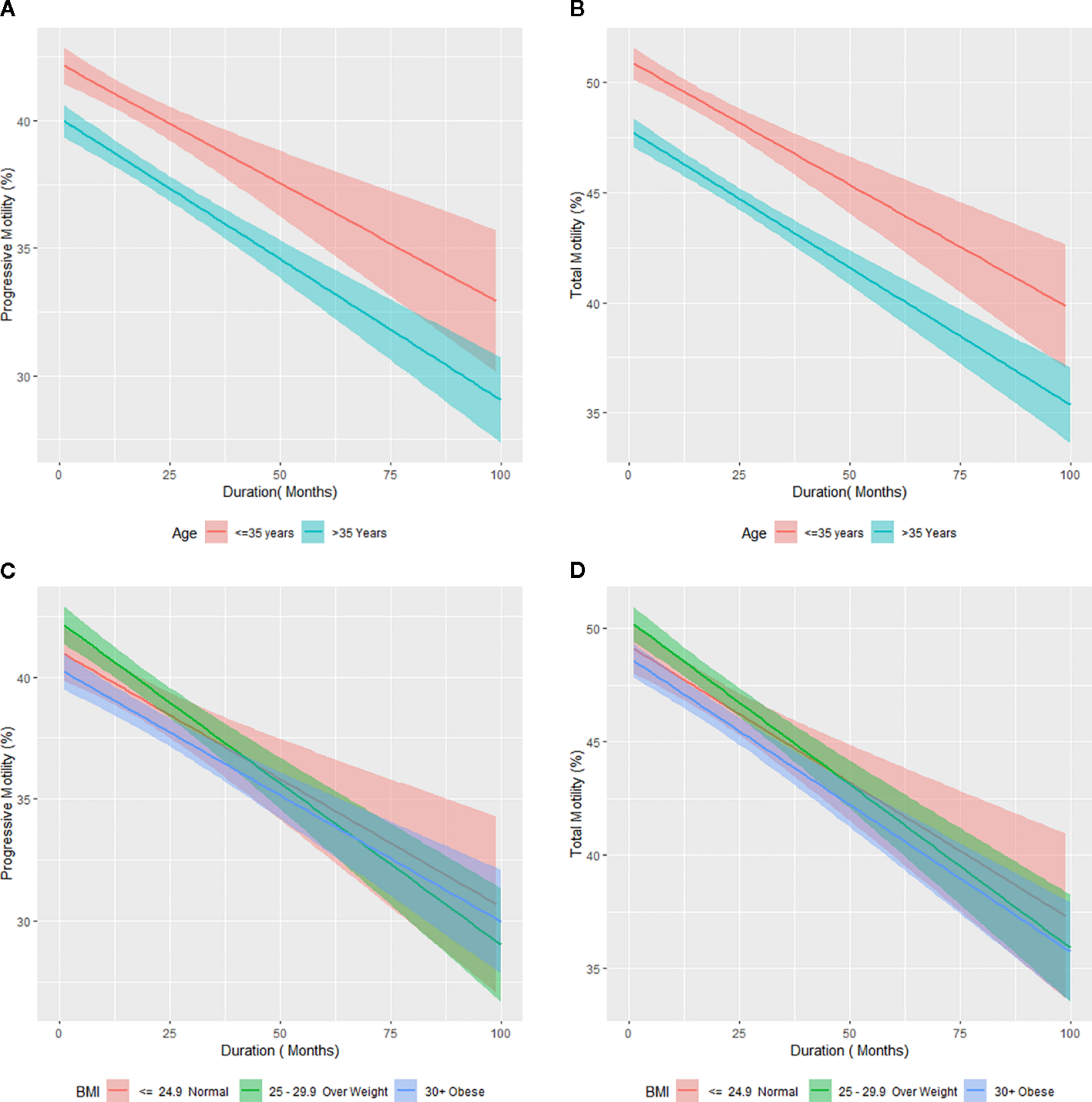
Effect of age and BMI on progressive and total motility over 100 months. **(A)** Age and progressive motility; **(B)** Age and total motility; **(C)** BMI and progressive motility; **(D)** BMI and total motility.

Patient age had a significant effect on sperm motility. Both progressive and total motility were significantly higher in the younger patient group (≤35 years) compared to the older group (>35 years) (progressive motility: 40.9 ± 19.8% vs 37.8 ± 19.4%; p<0.001) (total motility: 49.5 ± 19.9% vs 45.2 ± 20.0%; p<0.001) (**Figures 8A, B**). A distinct decline in motility was observed over the study duration in both age groups, although this trend did not reach statistical significance for both progressive motility and total motility parameters.

In contrast to the clear effect of age, no consistent or significant trends in progressive or total motility were observed across the different BMI categories over the course of the study period (**Figures 8C, D**, **Tables 6**, **7**).

**Table 6:** Regression analyses for the effect of age and BMI on progressive motility.

Parameter	Regression coefficient	95% confidence interval		P value
		Lower	Upper	
Intercept	45.798	43.229	48.367	<.001
Age	-2.058	-3.052	-1.063	<.001
BMI	-.478	-1.137	.182	.156
Duration (months)	-.094	-.184	-.004	.041
Age * Duration (months)	-.018	-.053	.018	.328
BMI * Duration (months)	.006	-.016	.027	.604

**Table 7:** Regression analyses for the effect of age and BMI on total motility.

Parameter	Regression coefficient	95% confidence interval		P value
		Lower	Upper	
Intercept	54.893	52.299	57.487	<.001
Age	-3.101	-4.107	-2.095	<.001
BMI	-.255	-.921	.412	.454
Duration (months)	-.093	-.184	-.002	.045
Age * Duration (months)	-.012	-.048	.024	.511
BMI * Duration (months)	-.002	-.024	.020	.838

## 4 Discussion

With the ongoing global discussion on declining fertility rates, particularly the documented decrease in semen quality, it becomes crucial to examine temporal trends in semen parameters while considering the potential influence of region, season, geography, and ethnicity. Such factors are essential for ensuring a precise and context-specific analysis of fertility trends. To generate robust, evidence-based statistics on global temporal trends in semen quality, it is important that data from as many countries as possible are represented. In this context, the current study examined temporal trends in semen parameters among Emirati men attending a fertility center in the UAE over 11 years.

Looking at the study of Mishra et al., where both fertile and infertile men were evaluated, their findings revealed that the trends in semen quality were largely comparable between the two groups (Mishra et al., 2018), suggesting that data obtained from men visiting fertility centers can, to a reasonable extent, reflect broader population trends.

Findings from the current study showed that while the mean values for all semen parameters remained above WHO lower reference values, notable fluctuations occurred during the study period. Semen volume and sperm count exhibited significant periods of fluctuation, whereas progressive and total motility displayed a consistent downward trend across the decade. Age and BMI influenced semen quality, though rates of temporal change were broadly similar across demographic groups.

There was a yearly decline in semen volume in this Emirati cohort between 2012 and 2016, after which values returned to levels observed at the start of the study and remained stable until 2022, although overall values remained within the normal range. Similar temporal declines and variability were observed by Iqbal et al. in a large retrospective cohort study from Saudi Arabia spanning 18 years, where semen volume gradually declined alongside other parameters (Ahmad et al., 2024). Additionally, studies from diverse populations, including those from Sicily (Italy) (Cannarella et al., 2021), South Africa (Akang et al., 2023), China (Li et al., 2023), and the USA (Punjani et al., 2022) have also reported a temporal decline in semen volume among men undergoing fertility evaluations. Similarly, we previously reported variations in semen parameters, including volume, among men from diverse ethnic backgrounds attending a fertility center in the UAE, highlighting how ethnicity and nationality can influence baseline semen profiles across populations (Omolaoye et al., 2024).

Sperm concentration and TSC exhibited fluctuating trends with distinct periods of decline and incline in the present study. Reports on temporal trends in sperm concentration and TSC are heterogeneous, with some populations showing no significant changes over time (Chen et al., 2003; Akang et al., 2023), while others reported a decline in these parameters (Lv et al., 2021; Li et al., 2023). The study of Iqbal et al. reported a significant downward trajectory in both sperm concentration and total sperm count in a Saudi cohort between 2004 and 2021 (Ahmad et al., 2024). While Aznavour et al. reported generally lower sperm counts in Middle Eastern men compared to Western populations, which contextualizes these fluctuations within a broader regional baseline, emphasizing the importance of considering population-specific reference ranges when interpreting trends (Aznavour et al., 2023).

Progressive and total motility consistently declined over the study period in our cohort. This trend is consistent with reports from multiple regions, although the extent and rate of decline vary significantly across different populations. For example, studies from New England between 1989 and 2000 demonstrated comparable gradual decreases in motility, indicating a widespread but moderate deterioration in sperm function over time (Chen et al., 2003). Similarly, data from a large fertility center in China analyzing over 49,000 men from 2015 to 2021 reported a measurable reduction in motility trends, though these declines were less pronounced than in Western cohorts, suggesting geographic and perhaps ethnic variability in susceptibility (Li et al., 2023). In contrast, a Sicilian cohort from 2011 to 2020 showed relatively stable motility, emphasizing regional differences shaped by lifestyle or environmental factors (Cannarella et al., 2021). On the contrary, studies from South Asia report more pronounced deteriorations; for example, Bangladeshi men attending a tertiary care hospital for fertility evaluations presented with significant declines in motility and other seminal parameters from 2000 to 2016 (Mahmud et al., 2018). Likewise, cohorts from India over 37 years (1979-2016) showed a sustained downward motility trend exceeding those in Western populations (Mishra et al., 2018). African studies from South Africa and Nigeria have similarly revealed decreases in motility amongst infertile men, underscoring that socioeconomic and environmental challenges may exacerbate reproductive health declines in developing regions (Akang et al., 2023).

The pronounced dip in semen parameters observed in 2016 represents a notable deviation from the gradual trends seen over the study period. While we did not directly measure physiological biomarkers, the overall decline may potentially be linked to multiple acute environmental stressors. Given that the region is an arid, subtropical desert climate characterized by sustained high temperatures, elevated ambient heat may increase scrotal temperature, leading to production of reactive oxidative species (ROS). ROS cause lipid peroxidation of sperm membranes, mitochondrial DNA damage, and nuclear DNA fragmentation, leading to dysfunctional sperm with reduced motility and viability (Yang et al., 2016). The mitochondria are particularly vulnerable as they generate ATP essential for sperm motility, and oxidative damage disrupts this energy production, further compromising sperm function (Li et al., 2021). Additionally, environmental pollutants commonly found in Middle Eastern urban areas, such as particulate matter, heavy metals, and endocrine-disrupting chemicals, may worsen oxidative stress and hormonal imbalances, further impacting sperm quality (Krzastek et al., 2021). Furthermore, obesity-related inflammation and hormonal imbalances amplify susceptibility to these environmental insults by reducing antioxidant defenses and impairing endocrine functions critical for spermatogenesis (Hammoud et al., 2008; Du Plessis et al., 2010). Although direct evidence from our cohort is lacking, emerging studies support that interactions of extreme heat, pollution, metabolic factors, and other stressors can acutely impair semen quality (Tesarik, 2025). Such multifactorial risks underscore the importance of targeted public health interventions to address male reproductive health amid growing environmental challenges.

Age had a consistent influence on semen quality in our cohort, with men over their mid-thirties showing modest declines in progressive and total motility, and volume. Whereas a marked increase was seen in sperm concentration and TSC in men over 35 years. Demirkol et al., identified the mid-thirties as a critical inflection point, beyond which consistent decreases in progressive motility, morphology, and vitality become statistically and clinically significant (Demirkol et al., 2021). Our findings are supported by Pino et al., who demonstrated accelerating declines in semen parameters, especially motility and volume, starting around age 35 and intensifying after 40 (Pino et al., 2020). Additionally, an earlier investigation by Stone et al. reported negligible changes in semen quality before 34 years but marked deterioration thereafter (Stone et al., 2013). The collective evidence positions the mid-thirties as a pivotal biological marker for reproductive aging in men, suggesting fertility evaluations and counselling should be tailored accordingly.

BMI exerted a significant influence on semen quality metrics in our cohort, with overweight men showing reduced semen volume, sperm concentration, and TSC relative to those with normal BMI. Whereas, obese men exhibited reduced volume, progressive and total motility, sperm concentration and TSC compared to the normal BMI group. This association is broadly supported by multiple studies worldwide (Sermondade et al., 2013; Wang et al., 2017; Ma et al., 2019). For instance, Wang et al. reported that high BMI was negatively associated with sperm concentration, total motile sperm count, and progressive motility in infertile men attending fertility clinics (Wang et al., 2017). Similarly, Ma et al.’s observational study highlighted that abnormal BMI strongly correlates with reductions in semen quality parameters, though the degree and specific parameters affected can vary across populations (Ma et al., 2019). These findings are consistent with the study of Sermondade et al. who showed a J-shaped relationship between BMI and oligozoospermia or azoospermia risk, with both underweight and obese men at increased risk (Sermondade et al., 2013). Notably, several studies find that while BMI markedly reduces baseline semen quality, the rate of longitudinal decline over time may not differ significantly between BMI categories. These data underscore the need for integrated metabolic health interventions to optimize baseline male reproductive potential before and during fertility treatment.

Our study, through its longitudinal approach and large cohort, offers a detailed temporal perspective on semen quality trends among Emirati men. While concluding that fluctuations in seminal parameters and their demographic determinants align with regional and international observations, the study’s retrospective nature, reduction of sample size over time, and single clinic-based sampling limit generalizability to the broader population. Additionally, detailed assessment of lifestyle, occupational exposures, male-factor infertility, and environmental factors was unavailable, constraining causal inference for observed trends. Future prospective studies integrating region-specific environmental, cultural, and lifestyle data into reproductive health programs and research are warranted to elucidate the complex determinants of male fertility in this context.

## 5 Conclusion

This study explored how semen parameters in Emirati men have changed over 11 years. Our results showed a notable year-to-year variation in key measures like semen volume, sperm concentration, total sperm count, and motility. These changes may be influenced by a combination of environmental, physiological, and lifestyle factors, a possibility that aligns with observations reported in other studies worldwide. Overall, these findings reflect just how complex and multifaceted male reproductive health is, and they highlight the need to always keep local context in mind when studying fertility trends. We recommend that future research and reproductive health initiatives focus on region-specific challenges by integrating environmental, cultural, and lifestyle factors to inform tailored strategies for improving male fertility outcomes.

## Data Availability

The original contributions presented in the study are included in the article/**Supplementary Material**. Further inquiries can be directed to the corresponding author.
