# Supplementary material for: Temporal trends in semen parameters among men attending a fertility center in the UAE (2012 – 2022): a retrospective study
Source: Front Physiol. 2026 Jun 10;17:1801736. doi: 10.3389/fphys.2026.1801736 (PMC13290661; doi:10.3389/fphys.2026.1801736)
Supplement: Supplementary file 1 [file DataSheet1.pdf]

Temporal trends in semen parameters among men attending a fertility center in the UAE from 2012 to 2022: A retrospective study

Temidayo S. Omolaoye[1], Jeyaseelan Lakshmanan[1], Amin Abu Hijleh[2], Irfan Aslam[3], Stefan S. Du Plessis[2*]
[1] College of Medicine, Mohammed Bin Rashid University of Medicine and Health Sciences, Dubai Health, Dubai, P.O.Box 505055, United Arab Emirates.
[2]Research and Graduate Studies, Mohammed Bin Rashid University of Medicine and Health Sciences, Dubai Health, Dubai, P.O.Box 505055, United Arab Emirates.
[3] HealthPlus Fertility Center, Abu Dhabi, P.O.Box 60843, United Arab Emirates.

Correspondence:
Stefan S. Du Plessis
Stefan.duplessis@dubaihealth.ae

Supplementary Table 1: Semen Volume

Date of Visit (Year)	Date of Visit (Year)	Mean Difference	Std. Error	Sig.	95% Confidence Interval	
					Lower Bound	Upper Bound
2012	2013	.04424	.21453	1.000	-.6676	.7561
	2014	.08259	.21419	1.000	-.6281	.7933
	2015	.29690	.21206	1.000	-.4067	1.0005
	2016	.81529*	.21193	.007	.1121	1.5185
	2017	.47062	.21088	1.000	-.2291	1.1703
	2018	.40169	.21081	1.000	-.2978	1.1012
	2019	.53350	.21080	.627	-.1659	1.2329
	2020	.58105	.21067	.320	-.1180	1.2801
	2021	.38313	.21019	1.000	-.3143	1.0805
	2022	.44071	.21329	1.000	-.2670	1.1484
2013	2012	-.04424	.21453	1.000	-.7561	.6676
	2014	.03835	.07551	1.000	-.2122	.2889
	2015	.25266*	.06923	.015	.0230	.4824
	2016	.77106*	.06884	<.001	.5426	.9995
	2017	.42638*	.06552	<.001	.2090	.6438
	2018	.35745*	.06532	<.001	.1407	.5742
	2019	.48926*	.06528	<.001	.2727	.7059
	2020	.53681*	.06486	<.001	.3216	.7520
	2021	.33889*	.06327	<.001	.1289	.5488
	2022	.39647*	.07291	<.001	.1546	.6384
2014	2012	-.08259	.21419	1.000	-.7933	.6281
	2013	-.03835	.07551	1.000	-.2889	.2122
	2015	.21431	.06816	.092	-.0119	.4405
	2016	.73270*	.06777	<.001	.5078	.9576
	2017	.38803*	.06439	<.001	.1744	.6017
	2018	.31910*	.06418	<.001	.1061	.5321
	2019	.45090*	.06415	<.001	.2381	.6637
	2020	.49845*	.06372	<.001	.2870	.7099
	2021	.30054*	.06210	<.001	.0945	.5066

	2022	.35812*	.07189	<.001	.1196	.5967
2015	2012	-.29690	.21206	1.000	-1.0005	.4067
	2013	-.25266*	.06923	.015	-.4824	-.0230
	2014	-.21431	.06816	.092	-.4405	.0119
	2016	.51839*	.06070	<.001	.3170	.7198
	2017	.17372	.05690	.125	-.0151	.3625
	2018	.10479	.05667	1.000	-.0832	.2928
	2019	.23660*	.05663	.002	.0487	.4245
	2020	.28415*	.05614	<.001	.0979	.4704
	2021	.08623	.05430	1.000	-.0939	.2664
	2022	.14381	.06527	1.000	-.0728	.3604
2016	2012	-.81529*	.21193	.007	-1.5185	-.1121
	2013	-.77106*	.06884	<.001	-.9995	-.5426
	2014	-.73270*	.06777	<.001	-.9576	-.5078
	2015	-.51839*	.06070	<.001	-.7198	-.3170
	2017	-.34467*	.05643	<.001	-.5319	-.1574
	2018	-.41361*	.05620	<.001	-.6001	-.2271
	2019	-.28180*	.05615	<.001	-.4681	-.0955
	2020	-.23425*	.05566	.001	-.4189	-.0496
	2021	-.43216*	.05381	<.001	-.6107	-.2536
	2022	-.37458*	.06486	<.001	-.5898	-.1594
2017	2012	-.47062	.21088	1.000	-1.1703	.2291
	2013	-.42638*	.06552	<.001	-.6438	-.2090
	2014	-.38803*	.06439	<.001	-.6017	-.1744
	2015	-.17372	.05690	.125	-.3625	.0151
	2016	.34467*	.05643	<.001	.1574	.5319
	2018	-.06893	.05207	1.000	-.2417	.1039
	2019	.06287	.05203	1.000	-.1097	.2355

	2020	.11043	.05150	1.000	-.0604	.2813
	2021	-.08749	.04948	1.000	-.2517	.0767
	2022	-.02991	.06133	1.000	-.2334	.1736
2018	2012	-.40169	.21081	1.000	-1.1012	.2978
	2013	-.35745*	.06532	<.001	-.5742	-.1407
	2014	-.31910*	.06418	<.001	-.5321	-.1061
	2015	-.10479	.05667	1.000	-.2928	.0832
	2016	.41361*	.05620	<.001	.2271	.6001
	2017	.06893	.05207	1.000	-.1039	.2417
	2019	.13181	.05177	.600	-.0400	.3036
	2020	.17936*	.05124	.026	.0093	.3494
	2021	-.01856	.04922	1.000	-.1819	.1447
	2022	.03902	.06111	1.000	-.1637	.2418
2019	2012	-.53350	.21080	.627	-1.2329	.1659
	2013	-.48926*	.06528	<.001	-.7059	-.2727
	2014	-.45090*	.06415	<.001	-.6637	-.2381
	2015	-.23660*	.05663	.002	-.4245	-.0487
	2016	.28180*	.05615	<.001	.0955	.4681
	2017	-.06287	.05203	1.000	-.2355	.1097
	2018	-.13181	.05177	.600	-.3036	.0400
	2020	.04755	.05119	1.000	-.1223	.2174
	2021	-.15036	.04917	.123	-.3135	.0128
	2022	-.09278	.06107	1.000	-.2954	.1098
2020	2012	-.58105	.21067	.320	-1.2801	.1180
	2013	-.53681*	.06486	<.001	-.7520	-.3216
	2014	-.49845*	.06372	<.001	-.7099	-.2870
	2015	-.28415*	.05614	<.001	-.4704	-.0979
	2016	.23425*	.05566	.001	.0496	.4189

	2017	-.11043	.05150	1.000	-.2813	.0604
	2018	-.17936*	.05124	.026	-.3494	-.0093
	2019	-.04755	.05119	1.000	-.2174	.1223
	2021	-.19792*	.04860	.003	-.3592	-.0366
	2022	-.14034	.06062	1.000	-.3415	.0608
2021	2012	-.38313	.21019	1.000	-1.0805	.3143
	2013	-.33889*	.06327	<.001	-.5488	-.1289
	2014	-.30054*	.06210	<.001	-.5066	-.0945
	2015	-.08623	.05430	1.000	-.2664	.0939
	2016	.43216*	.05381	<.001	.2536	.6107
	2017	.08749	.04948	1.000	-.0767	.2517
	2018	.01856	.04922	1.000	-.1447	.1819
	2019	.15036	.04917	.123	-.0128	.3135
	2020	.19792*	.04860	.003	.0366	.3592
	2022	.05758	.05892	1.000	-.1379	.2531
2022	2012	-.44071	.21329	1.000	-1.1484	.2670
	2013	-.39647*	.07291	<.001	-.6384	-.1546
	2014	-.35812*	.07189	<.001	-.5967	-.1196
	2015	-.14381	.06527	1.000	-.3604	.0728
	2016	.37458*	.06486	<.001	.1594	.5898
	2017	.02991	.06133	1.000	-.1736	.2334
	2018	-.03902	.06111	1.000	-.2418	.1637
	2019	.09278	.06107	1.000	-.1098	.2954
	2020	.14034	.06062	1.000	-.0608	.3415
	2021	-.05758	.05892	1.000	-.2531	.1379

*. The mean difference is significant at the 0.05 level.

Supplementary Table 2: Sperm Concentration and Total Sperm Count

Sperm Concentration						
Date of Visit (Year)	Date of Visit (Year)	Mean Difference (I-J)	Std. Error	Sig.	95% Confidence Interval	
					Lower Bound	Upper Bound
2012	2013	-11.10673	8.72541	1.000	-40.0582	17.8447
	2014	-12.81023	8.72754	1.000	-41.7688	16.1483
	2015	-6.41143	8.64399	1.000	-35.0927	22.2699
	2016	4.89655	8.63833	1.000	-23.7660	33.5591
	2017	-14.76357	8.60005	1.000	-43.2991	13.7719
	2018	-18.03465	8.59388	1.000	-46.5497	10.4804
	2019	-16.11406	8.59266	1.000	-44.6251	12.3969
	2020	-21.17761	8.58909	.753	-49.6768	7.3215
	2021	-25.78531	8.56966	.144	-54.2200	2.6494
	2022	-28.71342	8.68836	.052	-57.5419	.1151
2013	2012	11.10673	8.72541	1.000	-17.8447	40.0582
	2014	-1.70350	2.95897	1.000	-11.5216	8.1146
	2015	4.69530	2.70260	1.000	-4.2721	13.6627
	2016	16.00328*	2.68444	<.001	7.0961	24.9104
	2017	-3.65684	2.55860	1.000	-12.1464	4.8327
	2018	-6.92792	2.53776	.349	-15.3484	1.4925
	2019	-5.00733	2.53365	1.000	-13.4141	3.3995
	2020	-10.07088*	2.52151	.004	-18.4374	-1.7043
	2021	-14.67857*	2.45452	<.001	-22.8228	-6.5343
	2022	-17.60669*	2.84131	<.001	-27.0343	-8.1790
2014	2012	12.81023	8.72754	1.000	-16.1483	41.7688
	2013	1.70350	2.95897	1.000	-8.1146	11.5216
	2015	6.39880	2.70948	1.000	-2.5914	15.3890
	2016	17.70678*	2.69137	<.001	8.7766	26.6369

	2017	-1.95334	2.56587	1.000	-10.4671	6.5604
	2018	-5.22442	2.54509	1.000	-13.6692	3.2204
	2019	-3.30384	2.54099	1.000	-11.7350	5.1273
	2020	-8.36738	2.52889	.052	-16.7584	.0236
	2021	-12.97508*	2.46210	<.001	-21.1445	-4.8057
	2022	-15.90319*	2.84786	<.001	-25.3526	-6.4538
2015	2012	6.41143	8.64399	1.000	-22.2699	35.0927
	2013	-4.69530	2.70260	1.000	-13.6627	4.2721
	2014	-6.39880	2.70948	1.000	-15.3890	2.5914
	2016	11.30798*	2.40666	<.001	3.3225	19.2934
	2017	-8.35214*	2.26544	.013	-15.8690	-.8353
	2018	-11.62321*	2.24188	<.001	-19.0619	-4.1845
	2019	-9.70263*	2.23722	<.001	-17.1259	-2.2794
	2020	-14.76617*	2.22346	<.001	-22.1438	-7.3886
	2021	-19.37387*	2.14720	<.001	-26.4984	-12.2493
	2022	-22.30199*	2.58047	<.001	-30.8641	-13.7398
2016	2012	-4.89655	8.63833	1.000	-33.5591	23.7660
	2013	-16.00328*	2.68444	<.001	-24.9104	-7.0961
	2014	-17.70678*	2.69137	<.001	-26.6369	-8.7766
	2015	-11.30798*	2.40666	<.001	-19.2934	-3.3225
	2017	-19.66012*	2.24375	<.001	-27.1050	-12.2152
	2018	-22.93120*	2.21996	<.001	-30.2972	-15.5652
	2019	-21.01061*	2.21526	<.001	-28.3610	-13.6602
	2020	-26.07416*	2.20136	<.001	-33.3784	-18.7699
	2021	-30.68185*	2.12430	<.001	-37.7304	-23.6333
	2022	-33.60997*	2.56145	<.001	-42.1090	-25.1109
2017	2012	14.76357	8.60005	1.000	-13.7719	43.2991
	2013	3.65684	2.55860	1.000	-4.8327	12.1464
	2014	1.95334	2.56587	1.000	-6.5604	10.4671

	2015	8.35214*	2.26544	.013	.8353	15.8690
	2016	19.66012*	2.24375	<.001	12.2152	27.1050
	2018	-3.27107	2.06601	1.000	-10.1262	3.5841
	2019	-1.35049	2.06096	1.000	-8.1889	5.4879
	2020	-6.41403	2.04601	.095	-13.2028	.3748
	2021	-11.02173*	1.96287	<.001	-17.5346	-4.5088
	2022	-13.94984*	2.42924	<.001	-22.0102	-5.8895
2018	2012	18.03465	8.59388	1.000	-10.4804	46.5497
	2013	6.92792	2.53776	.349	-1.4925	15.3484
	2014	5.22442	2.54509	1.000	-3.2204	13.6692
	2015	11.62321*	2.24188	<.001	4.1845	19.0619
	2016	22.93120*	2.21996	<.001	15.5652	30.2972
	2017	3.27107	2.06601	1.000	-3.5841	10.1262
	2019	1.92058	2.03503	1.000	-4.8318	8.6729
	2020	-3.14296	2.01989	1.000	-9.8451	3.5592
	2021	-7.75066*	1.93563	.003	-14.1732	-1.3281
	2022	-10.67877*	2.40728	<.001	-18.6663	-2.6913
2019	2012	16.11406	8.59266	1.000	-12.3969	44.6251
	2013	5.00733	2.53365	1.000	-3.3995	13.4141
	2014	3.30384	2.54099	1.000	-5.1273	11.7350
	2015	9.70263*	2.23722	<.001	2.2794	17.1259
	2016	21.01061*	2.21526	<.001	13.6602	28.3610
	2017	1.35049	2.06096	1.000	-5.4879	8.1889
	2018	-1.92058	2.03503	1.000	-8.6729	4.8318
	2020	-5.06354	2.01473	.659	-11.7485	1.6215
	2021	-9.67124*	1.93024	<.001	-16.0759	-3.2666
	2022	-12.59935*	2.40295	<.001	-20.5725	-4.6262
2020	2012	21.17761	8.58909	.753	-7.3215	49.6768
	2013	10.07088*	2.52151	.004	1.7043	18.4374

	2014	8.36738	2.52889	.052	-.0236	16.7584
	2015	14.76617*	2.22346	<.001	7.3886	22.1438
	2016	26.07416*	2.20136	<.001	18.7699	33.3784
	2017	6.41403	2.04601	.095	-.3748	13.2028
	2018	3.14296	2.01989	1.000	-3.5592	9.8451
	2019	5.06354	2.01473	.659	-1.6215	11.7485
	2021	-4.60770	1.91427	.885	-10.9594	1.7440
	2022	-7.53581	2.39014	.089	-15.4665	.3948
2021	2012	25.78531	8.56966	.144	-2.6494	54.2200
	2013	14.67857*	2.45452	<.001	6.5343	22.8228
	2014	12.97508*	2.46210	<.001	4.8057	21.1445
	2015	19.37387*	2.14720	<.001	12.2493	26.4984
	2016	30.68185*	2.12430	<.001	23.6333	37.7304
	2017	11.02173*	1.96287	<.001	4.5088	17.5346
	2018	7.75066*	1.93563	.003	1.3281	14.1732
	2019	9.67124*	1.93024	<.001	3.2666	16.0759
	2020	4.60770	1.91427	.885	-1.7440	10.9594
	2022	-2.92811	2.31937	1.000	-10.6239	4.7677
2022	2012	28.71342	8.68836	.052	-.1151	57.5419
	2013	17.60669*	2.84131	<.001	8.1790	27.0343
	2014	15.90319*	2.84786	<.001	6.4538	25.3526
	2015	22.30199*	2.58047	<.001	13.7398	30.8641
	2016	33.60997*	2.56145	<.001	25.1109	42.1090
	2017	13.94984*	2.42924	<.001	5.8895	22.0102
	2018	10.67877*	2.40728	<.001	2.6913	18.6663
	2019	12.59935*	2.40295	<.001	4.6262	20.5725
	2020	7.53581	2.39014	.089	-.3948	15.4665
	2021	2.92811	2.31937	1.000	-4.7677	10.6239

*. The mean difference is significant at the 0.05 level.

Total sperm count						
Date of Visit (Year)	Date of Visit (Year)	Mean Difference (I-J)	Std. Error	Sig.	95% Confidence Interval	
					Lower Bound	Upper Bound
2012	2013	-7.96687	27.56697	1.000	-99.4359	83.5021
	2014	-3.81771	27.57249	1.000	-95.3050	87.6696
	2015	22.48588	27.31374	1.000	-68.1429	113.1146
	2016	77.66392	27.29622	.244	-12.9067	168.2346
	2017	21.81432	27.17864	1.000	-68.3662	111.9948
	2018	7.73868	27.15920	1.000	-82.3773	97.8547
	2019	20.88107	27.15519	1.000	-69.2216	110.9838
	2020	10.42485	27.14366	1.000	-79.6396	100.4893
	2021	-12.72178	27.08383	1.000	-102.5877	77.1441
	2022	-11.78159	27.45030	1.000	-102.8635	79.3003
2013	2012	7.96687	27.56697	1.000	-83.5021	99.4359
	2014	4.14916	9.25446	1.000	-26.5577	34.8560
	2015	30.45276*	8.45241	.017	2.4071	58.4984
	2016	85.63079*	8.39564	<.001	57.7735	113.4881
	2017	29.78120*	8.00508	.011	3.2198	56.3426
	2018	15.70556	7.93884	1.000	-10.6360	42.0471
	2019	28.84794*	7.92512	.015	2.5519	55.1440
	2020	18.39172	7.88552	1.000	-7.7730	44.5564
	2021	-4.75490	7.67702	1.000	-30.2278	20.7179
	2022	-3.81472	8.88381	1.000	-33.2918	25.6623
2014	2012	3.81771	27.57249	1.000	-87.6696	95.3050
	2013	-4.14916	9.25446	1.000	-34.8560	26.5577
	2015	26.30359	8.47039	.105	-1.8017	54.4089
	2016	81.48163*	8.41375	<.001	53.5643	109.3990
	2017	25.63203	8.02406	.077	-.9923	52.2564
	2018	11.55639	7.95799	1.000	-14.8487	37.9615

	2019	24.69877	7.94430	.103	-1.6609	51.0585
	2020	14.24256	7.90480	1.000	-11.9861	40.4712
	2021	-8.90407	7.69682	1.000	-34.4426	16.6345
	2022	-7.96388	8.90092	1.000	-37.4977	21.5700
2015	2012	-22.48588	27.31374	1.000	-113.1146	68.1429
	2013	-30.45276*	8.45241	.017	-58.4984	-2.4071
	2014	-26.30359	8.47039	.105	-54.4089	1.8017
	2016	55.17804*	7.52259	<.001	30.2176	80.1385
	2017	-.67156	7.08406	1.000	-24.1769	22.8338
	2018	-14.74720	7.00912	1.000	-38.0039	8.5095
	2019	-1.60482	6.99357	1.000	-24.8100	21.6003
	2020	-12.06103	6.94867	1.000	-35.1172	10.9951
	2021	-35.20766*	6.71113	<.001	-57.4756	-12.9397
	2022	-34.26747*	8.06379	.001	-61.0236	-7.5113
2016	2012	-77.66392	27.29622	.244	-168.2346	12.9067
	2013	-85.63079*	8.39564	<.001	-113.4881	-57.7735
	2014	-81.48163*	8.41375	<.001	-109.3990	-53.5643
	2015	-55.17804*	7.52259	<.001	-80.1385	-30.2176
	2017	-55.84960*	7.01623	<.001	-79.1299	-32.5693
	2018	-69.92524*	6.94056	<.001	-92.9545	-46.8960
	2019	-56.78286*	6.92486	<.001	-79.7600	-33.8057
	2020	-67.23907*	6.87951	<.001	-90.0657	-44.4124
	2021	-90.38570*	6.63949	<.001	-112.4159	-68.3554
	2022	-89.44551*	8.00426	<.001	-116.0042	-62.8868
2017	2012	-21.81432	27.17864	1.000	-111.9948	68.3662
	2013	-29.78120*	8.00508	.011	-56.3426	-3.2198
	2014	-25.63203	8.02406	.077	-52.2564	.9923
	2015	.67156	7.08406	1.000	-22.8338	24.1769
	2016	55.84960*	7.01623	<.001	32.5693	79.1299

	2018	-14.07564	6.46265	1.000	-35.5191	7.3679
	2019	-.93326	6.44579	1.000	-22.3208	20.4543
	2020	-11.38948	6.39704	1.000	-32.6153	9.8363
	2021	-34.53610*	6.13818	<.001	-54.9030	-14.1692
	2022	-33.59592*	7.59360	<.001	-58.7920	-8.3999
2018	2012	-7.73868	27.15920	1.000	-97.8547	82.3773
	2013	-15.70556	7.93884	1.000	-42.0471	10.6360
	2014	-11.55639	7.95799	1.000	-37.9615	14.8487
	2015	14.74720	7.00912	1.000	-8.5095	38.0039
	2016	69.92524*	6.94056	<.001	46.8960	92.9545
	2017	14.07564	6.46265	1.000	-7.3679	35.5191
	2019	13.14238	6.36334	1.000	-7.9716	34.2564
	2020	2.68617	6.31396	1.000	-18.2640	23.6363
	2021	-20.46046*	6.05155	.040	-40.5399	-.3810
	2022	-19.52028	7.52374	.522	-44.4845	5.4440
2019	2012	-20.88107	27.15519	1.000	-110.9838	69.2216
	2013	-28.84794*	7.92512	.015	-55.1440	-2.5519
	2014	-24.69877	7.94430	.103	-51.0585	1.6609
	2015	1.60482	6.99357	1.000	-21.6003	24.8100
	2016	56.78286*	6.92486	<.001	33.8057	79.7600
	2017	.93326	6.44579	1.000	-20.4543	22.3208
	2018	-13.14238	6.36334	1.000	-34.2564	7.9716
	2020	-10.45622	6.29669	1.000	-31.3491	10.4366
	2021	-33.60284*	6.03353	<.001	-53.6225	-13.5832
	2022	-32.66266*	7.50926	<.001	-57.5789	-7.7464
2020	2012	-10.42485	27.14366	1.000	-100.4893	79.6396
	2013	-18.39172	7.88552	1.000	-44.5564	7.7730
	2014	-14.24256	7.90480	1.000	-40.4712	11.9861
	2015	12.06103	6.94867	1.000	-10.9951	35.1172

	2016	67.23907*	6.87951	<.001	44.4124	90.0657
	2017	11.38948	6.39704	1.000	-9.8363	32.6153
	2018	-2.68617	6.31396	1.000	-23.6363	18.2640
	2019	10.45622	6.29669	1.000	-10.4366	31.3491
	2021	-23.14663*	5.98143	.006	-42.9934	-3.2999
	2022	-22.20644	7.46746	.162	-46.9840	2.5711
2021	2012	12.72178	27.08383	1.000	-77.1441	102.5877
	2013	4.75490	7.67702	1.000	-20.7179	30.2278
	2014	8.90407	7.69682	1.000	-16.6345	34.4426
	2015	35.20766*	6.71113	<.001	12.9397	57.4756
	2016	90.38570*	6.63949	<.001	68.3554	112.4159
	2017	34.53610*	6.13818	<.001	14.1692	54.9030
	2018	20.46046*	6.05155	.040	.3810	40.5399
	2019	33.60284*	6.03353	<.001	13.5832	53.6225
	2020	23.14663*	5.98143	.006	3.2999	42.9934
	2022	.94018	7.24694	1.000	-23.1056	24.9860
2022	2012	11.78159	27.45030	1.000	-79.3003	102.8635
	2013	3.81472	8.88381	1.000	-25.6623	33.2918
	2014	7.96388	8.90092	1.000	-21.5700	37.4977
	2015	34.26747*	8.06379	.001	7.5113	61.0236
	2016	89.44551*	8.00426	<.001	62.8868	116.0042
	2017	33.59592*	7.59360	<.001	8.3999	58.7920
	2018	19.52028	7.52374	.522	-5.4440	44.4845
	2019	32.66266*	7.50926	<.001	7.7464	57.5789
	2020	22.20644	7.46746	.162	-2.5711	46.9840
	2021	-.94018	7.24694	1.000	-24.9860	23.1056

*. The mean difference is significant at the 0.05 level.

Supplementary Table 3. Progressive and Total Motility

Progressive Motility						
Date of Visit (Year)	Date of Visit (Year)	Mean Difference (I-J)	Std. Error	Sig.	95% Confidence Interval	
					Lower Bound	Upper Bound
2012	2013	-3.8106	3.1362	1.000	-14.217	6.596
	2014	-6.3784	3.1328	1.000	-16.773	4.016
	2015	.1396	3.1072	1.000	-10.170	10.449
	2016	2.2248	3.1038	1.000	-8.074	12.523
	2017	2.9770	3.0903	1.000	-7.277	13.231
	2018	4.5164	3.0875	1.000	-5.728	14.761
	2019	5.2631	3.0872	1.000	-4.981	15.507
	2020	8.5765	3.0861	.300	-1.663	18.816
	2021	5.9937	3.0796	1.000	-4.225	16.212
	2022	7.5443	3.1193	.858	-2.806	17.894
2013	2012	3.8106	3.1362	1.000	-6.596	14.217
	2014	-2.5678	1.0418	.755	-6.025	.889
	2015	3.9502*	.9620	.002	.758	7.142
	2016	6.0354*	.9509	<.001	2.880	9.191
	2017	6.7876*	.9061	<.001	3.781	9.794
	2018	8.3270*	.8965	<.001	5.352	11.302
	2019	9.0737*	.8955	<.001	6.102	12.045
	2020	12.3871*	.8916	<.001	9.429	15.345
	2021	9.8043*	.8687	<.001	6.922	12.687
	2022	11.3549*	1.0003	<.001	8.036	14.674
2014	2012	6.3784	3.1328	1.000	-4.016	16.773
	2013	2.5678	1.0418	.755	-.889	6.025
	2015	6.5181*	.9508	<.001	3.363	9.673
	2016	8.6032*	.9396	<.001	5.486	11.721
	2017	9.3554*	.8942	<.001	6.388	12.323

	2018	10.8948*	.8845	<.001	7.960	13.830
	2019	11.6415*	.8834	<.001	8.710	14.573
	2020	14.9549*	.8795	<.001	12.037	17.873
	2021	12.3721*	.8563	<.001	9.531	15.213
	2022	13.9227*	.9896	<.001	10.639	17.206
2015	2012	-.1396	3.1072	1.000	-10.449	10.170
	2013	-3.9502*	.9620	.002	-7.142	-.758
	2014	-6.5181*	.9508	<.001	-9.673	-3.363
	2016	2.0852	.8503	.781	-.736	4.906
	2017	2.8374*	.7998	.021	.183	5.491
	2018	4.3767*	.7889	<.001	1.759	6.994
	2019	5.1235*	.7878	<.001	2.510	7.737
	2020	8.4368*	.7833	<.001	5.838	11.036
	2021	5.8540*	.7572	<.001	3.341	8.367
	2022	7.4046*	.9052	<.001	4.401	10.408
2016	2012	-2.2248	3.1038	1.000	-12.523	8.074
	2013	-6.0354*	.9509	<.001	-9.191	-2.880
	2014	-8.6032*	.9396	<.001	-11.721	-5.486
	2015	-2.0852	.8503	.781	-4.906	.736
	2017	.7522	.7865	1.000	-1.858	3.362
	2018	2.2916	.7754	.172	-.281	4.865
	2019	3.0383*	.7743	.005	.469	5.607
	2020	6.3517*	.7698	<.001	3.798	8.906
	2021	3.7688*	.7432	<.001	1.303	6.235
	2022	5.3195*	.8935	<.001	2.355	8.284
2017	2012	-2.9770	3.0903	1.000	-13.231	7.277
	2013	-6.7876*	.9061	<.001	-9.794	-3.781
	2014	-9.3554*	.8942	<.001	-12.323	-6.388
	2015	-2.8374*	.7998	.021	-5.491	-.183

	2016	-.7522	.7865	1.000	-3.362	1.858
	2018	1.5394	.7198	1.000	-.849	3.928
	2019	2.2861	.7185	.081	-.098	4.670
	2020	5.5995*	.7136	<.001	3.232	7.967
	2021	3.0167*	.6849	<.001	.744	5.289
	2022	4.5673*	.8456	<.001	1.762	7.373
2018	2012	-4.5164	3.0875	1.000	-14.761	5.728
	2013	-8.3270*	.8965	<.001	-11.302	-5.352
	2014	-10.8948*	.8845	<.001	-13.830	-7.960
	2015	-4.3767*	.7889	<.001	-6.994	-1.759
	2016	-2.2916	.7754	.172	-4.865	.281
	2017	-1.5394	.7198	1.000	-3.928	.849
	2019	.7467	.7063	1.000	-1.597	3.090
	2020	4.0601*	.7014	<.001	1.733	6.387
	2021	1.4773	.6721	1.000	-.753	3.707
	2022	3.0279*	.8353	.016	.256	5.799
2019	2012	-5.2631	3.0872	1.000	-15.507	4.981
	2013	-9.0737*	.8955	<.001	-12.045	-6.102
	2014	-11.6415*	.8834	<.001	-14.573	-8.710
	2015	-5.1235*	.7878	<.001	-7.737	-2.510
	2016	-3.0383*	.7743	.005	-5.607	-.469
	2017	-2.2861	.7185	.081	-4.670	.098
	2018	-.7467	.7063	1.000	-3.090	1.597
	2020	3.3134*	.7001	<.001	.990	5.636
	2021	.7306	.6707	1.000	-1.495	2.956
	2022	2.2812	.8342	.344	-.487	5.049
2020	2012	-8.5765	3.0861	.300	-18.816	1.663
	2013	-12.3871*	.8916	<.001	-15.345	-9.429
	2014	-14.9549*	.8795	<.001	-17.873	-12.037

	2015	-8.4368*	.7833	<.001	-11.036	-5.838
	2016	-6.3517*	.7698	<.001	-8.906	-3.798
	2017	-5.5995*	.7136	<.001	-7.967	-3.232
	2018	-4.0601*	.7014	<.001	-6.387	-1.733
	2019	-3.3134*	.7001	<.001	-5.636	-.990
	2021	-2.5828*	.6655	.006	-4.791	-.375
	2022	-1.0322	.8300	1.000	-3.786	1.722
2021	2012	-5.9937	3.0796	1.000	-16.212	4.225
	2013	-9.8043*	.8687	<.001	-12.687	-6.922
	2014	-12.3721*	.8563	<.001	-15.213	-9.531
	2015	-5.8540*	.7572	<.001	-8.367	-3.341
	2016	-3.7688*	.7432	<.001	-6.235	-1.303
	2017	-3.0167*	.6849	<.001	-5.289	-.744
	2018	-1.4773	.6721	1.000	-3.707	.753
	2019	-.7306	.6707	1.000	-2.956	1.495
	2020	2.5828*	.6655	.006	.375	4.791
	2022	1.5506	.8054	1.000	-1.122	4.223
2022	2012	-7.5443	3.1193	.858	-17.894	2.806
	2013	-11.3549*	1.0003	<.001	-14.674	-8.036
	2014	-13.9227*	.9896	<.001	-17.206	-10.639
	2015	-7.4046*	.9052	<.001	-10.408	-4.401
	2016	-5.3195*	.8935	<.001	-8.284	-2.355
	2017	-4.5673*	.8456	<.001	-7.373	-1.762
	2018	-3.0279*	.8353	.016	-5.799	-.256
	2019	-2.2812	.8342	.344	-5.049	.487
	2020	1.0322	.8300	1.000	-1.722	3.786
	2021	-1.5506	.8054	1.000	-4.223	1.122

*. The mean difference is significant at the 0.05 level.

Total Motility

Date of Visit (Year)	Date of Visit (Year)	Mean Difference (I-J)	Std. Error	Sig.	95% Confidence Interval	
					Lower Bound	Upper Bound
2012	2013	-5.1978	3.1451	1.000	-15.634	5.238
	2014	-9.4313	3.1404	.147	-19.851	.989
	2015	-2.6122	3.1152	1.000	-12.948	7.724
	2016	-.2193	3.1113	1.000	-10.543	10.104
	2017	3.8139	3.0978	1.000	-6.465	14.093
	2018	5.0866	3.0950	1.000	-5.183	15.356
	2019	6.4959	3.0947	1.000	-3.772	16.764
	2020	9.7698	3.0936	.088	-.495	20.035
	2021	7.3246	3.0870	.972	-2.918	17.568
	2022	7.9895	3.1268	.584	-2.385	18.364
2013	2012	5.1978	3.1451	1.000	-5.238	15.634
	2014	-4.2335*	1.0483	.003	-7.712	-.755
	2015	2.5856	.9702	.424	-.634	5.805
	2016	4.9784*	.9578	<.001	1.800	8.157
	2017	9.0117*	.9130	<.001	5.982	12.041
	2018	10.2844*	.9034	<.001	7.287	13.282
	2019	11.6937*	.9023	<.001	8.700	14.688
	2020	14.9676*	.8985	<.001	11.986	17.949
	2021	12.5224*	.8757	<.001	9.617	15.428
	2022	13.1872*	1.0069	<.001	9.846	16.528
2014	2012	9.4313	3.1404	.147	-.989	19.851
	2013	4.2335*	1.0483	.003	.755	7.712
	2015	6.8191*	.9547	<.001	3.651	9.987
	2016	9.2120*	.9421	<.001	6.086	12.338
	2017	13.2452*	.8965	<.001	10.271	16.220
	2018	14.5179*	.8867	<.001	11.576	17.460
	2019	15.9272*	.8856	<.001	12.989	18.866

	2020	19.2011*	.8817	<.001	16.276	22.127
	2021	16.7560*	.8585	<.001	13.908	19.604
	2022	17.4208*	.9920	<.001	14.129	20.712
2015	2012	2.6122	3.1152	1.000	-7.724	12.948
	2013	-2.5856	.9702	.424	-5.805	.634
	2014	-6.8191*	.9547	<.001	-9.987	-3.651
	2016	2.3928	.8543	.281	-.442	5.227
	2017	6.4261*	.8038	<.001	3.759	9.093
	2018	7.6988*	.7928	<.001	5.068	10.330
	2019	9.1081*	.7916	<.001	6.482	11.735
	2020	12.3820*	.7872	<.001	9.770	14.994
	2021	9.9368*	.7611	<.001	7.411	12.462
	2022	10.6016*	.9090	<.001	7.585	13.618
2016	2012	.2193	3.1113	1.000	-10.104	10.543
	2013	-4.9784*	.9578	<.001	-8.157	-1.800
	2014	-9.2120*	.9421	<.001	-12.338	-6.086
	2015	-2.3928	.8543	.281	-5.227	.442
	2017	4.0332*	.7888	<.001	1.416	6.650
	2018	5.3059*	.7776	<.001	2.726	7.886
	2019	6.7152*	.7763	<.001	4.139	9.291
	2020	9.9891*	.7719	<.001	7.428	12.550
	2021	7.5440*	.7453	<.001	5.071	10.017
	2022	8.2088*	.8958	<.001	5.236	11.181
2017	2012	-3.8139	3.0978	1.000	-14.093	6.465
	2013	-9.0117*	.9130	<.001	-12.041	-5.982
	2014	-13.2452*	.8965	<.001	-16.220	-10.271
	2015	-6.4261*	.8038	<.001	-9.093	-3.759
	2016	-4.0332*	.7888	<.001	-6.650	-1.416
	2018	1.2727	.7218	1.000	-1.122	3.668

	2019	2.6820*	.7204	.011	.292	5.072
	2020	5.9559*	.7156	<.001	3.581	8.330
	2021	3.5108*	.6867	<.001	1.232	5.789
	2022	4.1756*	.8478	<.001	1.363	6.988
2018	2012	-5.0866	3.0950	1.000	-15.356	5.183
	2013	-10.2844*	.9034	<.001	-13.282	-7.287
	2014	-14.5179*	.8867	<.001	-17.460	-11.576
	2015	-7.6988*	.7928	<.001	-10.330	-5.068
	2016	-5.3059*	.7776	<.001	-7.886	-2.726
	2017	-1.2727	.7218	1.000	-3.668	1.122
	2019	1.4093	.7082	1.000	-.940	3.759
	2020	4.6832*	.7033	<.001	2.350	7.017
	2021	2.2380*	.6739	.050	.002	4.474
	2022	2.9029*	.8374	.029	.124	5.681
2019	2012	-6.4959	3.0947	1.000	-16.764	3.772
	2013	-11.6937*	.9023	<.001	-14.688	-8.700
	2014	-15.9272*	.8856	<.001	-18.866	-12.989
	2015	-9.1081*	.7916	<.001	-11.735	-6.482
	2016	-6.7152*	.7763	<.001	-9.291	-4.139
	2017	-2.6820*	.7204	.011	-5.072	-.292
	2018	-1.4093	.7082	1.000	-3.759	.940
	2020	3.2739*	.7019	<.001	.945	5.603
	2021	.8288	.6724	1.000	-1.402	3.060
	2022	1.4936	.8362	1.000	-1.281	4.268
2020	2012	-9.7698	3.0936	.088	-20.035	.495
	2013	-14.9676*	.8985	<.001	-17.949	-11.986
	2014	-19.2011*	.8817	<.001	-22.127	-16.276
	2015	-12.3820*	.7872	<.001	-14.994	-9.770
	2016	-9.9891*	.7719	<.001	-12.550	-7.428

	2017	-5.9559*	.7156	<.001	-8.330	-3.581
	2018	-4.6832*	.7033	<.001	-7.017	-2.350
	2019	-3.2739*	.7019	<.001	-5.603	-.945
	2021	-2.4451*	.6673	.014	-4.659	-.231
	2022	-1.7803	.8321	1.000	-4.541	.981
2021	2012	-7.3246	3.0870	.972	-17.568	2.918
	2013	-12.5224*	.8757	<.001	-15.428	-9.617
	2014	-16.7560*	.8585	<.001	-19.604	-13.908
	2015	-9.9368*	.7611	<.001	-12.462	-7.411
	2016	-7.5440*	.7453	<.001	-10.017	-5.071
	2017	-3.5108*	.6867	<.001	-5.789	-1.232
	2018	-2.2380*	.6739	.050	-4.474	-.002
	2019	-.8288	.6724	1.000	-3.060	1.402
	2020	2.4451*	.6673	.014	.231	4.659
	2022	.6648	.8074	1.000	-2.014	3.344
2022	2012	-7.9895	3.1268	.584	-18.364	2.385
	2013	-13.1872*	1.0069	<.001	-16.528	-9.846
	2014	-17.4208*	.9920	<.001	-20.712	-14.129
	2015	-10.6016*	.9090	<.001	-13.618	-7.585
	2016	-8.2088*	.8958	<.001	-11.181	-5.236
	2017	-4.1756*	.8478	<.001	-6.988	-1.363
	2018	-2.9029*	.8374	.029	-5.681	-.124
	2019	-1.4936	.8362	1.000	-4.268	1.281
	2020	1.7803	.8321	1.000	-.981	4.541
	2021	-.6648	.8074	1.000	-3.344	2.014

*. The mean difference is significant at the 0.05 level.
